# VEGFR3 as a novel modulator for PAH

**DOI:** 10.18632/oncotarget.21295

**Published:** 2017-09-28

**Authors:** Heon-Woo Lee, Suk-Won Jin

**Affiliations:** Suk-Won Jin: Yale Cardiovascular Research Center, Department of Internal Medicine, Yale University School of Medicine, New Haven, CT, USA; School of Life Sciences and Cell Logistics Research Center, Gwangju Institute of Science and Technology, Gwangju, Republic of Korea

**Keywords:** BMPR2, VEGFR3, pulmonary hypertension, signaling

Pulmonary artery hypertension (PAH) is a rare lung disorder in which the arteries that carry blood from the heart to the lungs progressively narrow down, making it difficult for blood to flow through the vessels. In many cases, PAH leads to right ventricular failure. The recent estimate of 1-, 3-, and 5-year survival rates from the time of diagnosis were estimated to be 87.7%, 72.1% , and 60.3%, which are substantially lower than breast cancer or colon cancers [1]. Significant advances in the understanding of PAH over the last two decades have led to the introduction of multiple therapeutic target for PAH, including oral anticoagulation, diuretics, oxygen supplementation, endothelin receptor antagonists, and phosphodiesterase type 5 inhibitors. Despite 10 approved drugs [2], many PAH patients still suffer from a significantly high mortality rate since current treatment is focused on relieving acute symptoms rather than primary causes of PAH.

Arguably the most critical signaling pathway for the onset and progression of PAH is the Bone Morphogenetic Protein (BMP) signaling [3]. Mutation in one of the major receptors for BMP signaling, namely BMPR2 have been identified in both idiopathic and familial PAH patients, substantiating the importance of BMP signaling for the pathophysiology of PAH. Mutations causing loss of BMPR2 function are found in 75%-80% of familial and approximately 20% of IPAH patient. It was observed that PAH caused by BMPR2 mutation is a familial disease transmitted in an autosomal dominant manner. But, in spite of the importance of BMPR2 in PAH patients and an autosomal dominant inheritance, BMPR2 do not affect all mutation carriers due to reduced penetrance even within a PAH patient family. True estimates of penetrance will probably vary with the nature of the underlying mutation, but on average is expected to be 20–30% [2]. Thus, many patients who carry the disease gene do not manifest clinical PAH. In addition, BMPR2 knockout rodents do not develop PAH and required additional ‘hits’ or triggers (like hypoxia, monocrotaline or Sugen5416) to induce PAH. Therefore, it has been speculated that additional modulators may determine the penetrance of the PAH [4, 5].

Similar to other signaling pathways mediated by surface receptors, the amplitude and duration of BMP signaling is regulated by the endocytosis [6]. Therefore, it is conceivable that the alteration of receptor endocytosis may contribute to the pathogenesis of PAH. In the recent issue of *Circulation*, Hwangbo and colleagues reported that VEGFR3, a tyrosine-protein kinase that acts as a receptor for VEGF-C and VEGF-D, may function as a potential modifier for BMP signaling in PAH setting by modulating the endocytosis of BMPR2 [7]. VEGFR3 has been regarded as a potential therapeutic target for various disease due to its specific expression pattern in endothelial cells and lymphatic endothelial cells. Using a number of experimental systems, Hwangbo and colleagues elegantly demonstrated that VEGFR3-BMPR2 interaction is crucial to promote BMPR2 endocytosis and to induce phosphorylation of SMADs. In addition, endothelial specific inducible deletion of VEGFR3 (VEGF3^floxed/ floxed^; Cadherin5-Cre^ERT2^) in mice led to exacerbated pulmonary hypertension after exposure to chronic hypoxia and impaired BMP signaling responses compared to their phenotypic wild-type littermates, further corroborating the critical role of VEGFR3 in BMP signaling. Consistent with these data, they found pulmonary arterial endothelial cells (PAECs) isolated from PAH patients were insensitive to BMP stimulation also displayed significantly decreased level of VEGFR3 expression. Interestingly, these cells became responsive to BMP stimulation upon forced expression of VEGFR3, raising the possibility that manipulation of VEGFR3 in PAECs may be used to restore BMP responsiveness in PAECs in PAH setting. Further investigation on the molecular basis of BMPR2-VEGFR3 interaction may shed light on intricate interplay among diverse signaling nodes during the pathogenesis of PAH.

**Figure 1:**
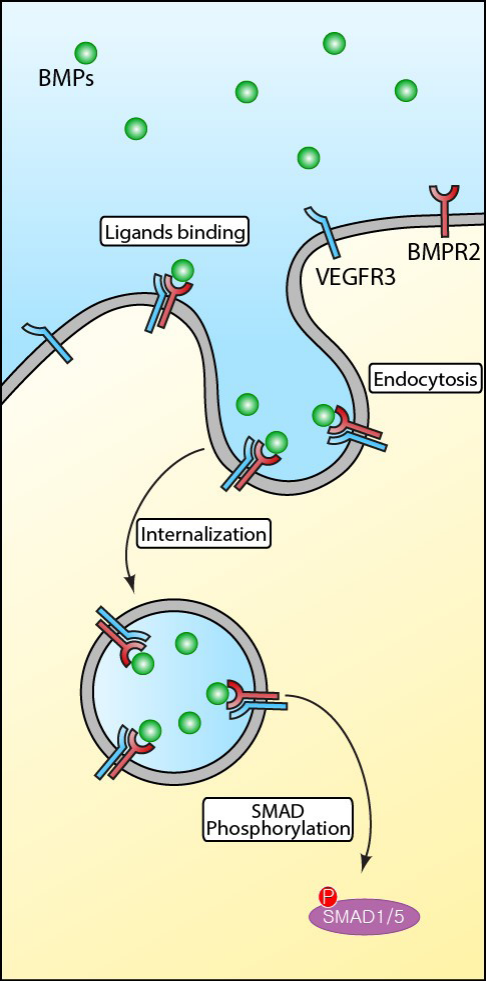
Interaction between BMPR2 and VEGFR3 is critical for the ligand induced endocytosis of BMP receptors in endothelial cells BMPR2 is located at the endothelial cell surface membrane in the resting states. Upon ligand binding, BMPR2 may recruit VEGFR3 and undergoes Clathrin-mediated endocytosis to induce downstream signaling cascades such as phosphorylation of R-SMADs (SMAD1 and SMAD5).
